# A Case of Infanticide

**Published:** 1853-01

**Authors:** W. L. Sutton

**Affiliations:** Georgetown, Ky.


Art. II.—*A Case of Infanticide.* By W. L. Sutton, M. D. of Georgetown, Ky.

As Medical Jurisprudence has engaged but little attention among physicians in the West, I think proper to lay the following case before them.

I was required by the Coroner of Scott county to attend and aid the jury of inquest, to be held upon the body of an infant found dead at the house of P. Willging in

Georgetown. We found a black female child 19 inches long, weighing 7 pounds averdupois, having the unctuous matter usually found upon newly born babes. From the top of the head to the umbilicus measured 11 inches—from umbilicus to soles of the feet 8 inches. Umbilical cord torn off 7 inches from navel. Hair and nails well developed. *Chest* well expanded. No signs of injury externally. Upon cutting the skin and cellular membrane the blood flowed. *Heart* not entirely covered by the lungs, but nearly so. Lungs, pale pink color; presence of air in them readily detected by pressure; being cut into, blood followed the knife. Being taken out and put into water, with the heart attached, they floated buoyantly.

| | | | |
|---|---|---|---|
| Weight of lungs and heart - - | 1097 grs. Troy | Weight of heart and lungs to body - | 1 to 41.61 |
| " of lungs alone | 770.5 " " | " of heart and lungs alone to body | 1 to 149.9– |
| " of heart alone | 326.5 " " | " of lungs alone to body | 1 to 62.2– |
| | | " of heart to lungs | 1 to 2.36– |

Bladder contracted. On each side of the sagittal suture, where it extends into the os frontis, were numerous points of extravasated blood. Brain uninjured. In handling the child, a fluid, stained with feces, was observed in the mouth.

Sept. 23. It was in evidence before the court of inquiry, it was proved, that Susan, the supposed mother, had always denied that she was pregnant. After delivery she denied that any delivery had occurred.

The child was found lying on the right side, face inclining downwards, in the vault of a privy, about half covered with water; little or no blood about it. The vault about 18 inches deep from the seat. The floor of the privy had been washed before the witness arrived, but was said to have contained a great deal of blood.

Matilda, a negro woman, testified that she took the child from the privy. Knew that the child was Susan's, because she took the afterbirth from her. Susan subsequently acknowledged the birth of the child, but said it took place whilst she was sitting on the hole in the privy. Susan was committed for further trial; bailed, and then sent down the river.

I have thought proper to report this case, among other reasons, because a highly respectable attorney gave it as his opinion that the testimony did not warrant the commitment. The Commonwealth's attorney also, at the ensuing term of court, discouraged the finding of a true bill by the Grand Jury. To be sure the woman was then beyond the bounds of this commonwealth—but her surety was not.

The first remark which I shall make is that the general appearance of the child warranted the belief that it was born at full term,—the length, weight, and developement of the hair and nails; yet the navel was full an inch and a half below the middle of the body—contrary to the rule laid down by Prof. Dewees. The proportional weight between the heart and lungs separately and together, and that of the body—also of that of the heart to the lungs lead to no conclusion worth notice.

I paid no attention to the foramen ovale—the ductus arteriosus or ductus venosus—because the recency of birth precluded the idea of any appreciable change having taken place.

But the question is, was this a case of infanticide. As the birth had unquestionably occurred within a very few hours, and as the lungs were fully inflated, there is no reason to doubt that the child was born alive. It would seem also that it had made several inspirations. I come to this conclusion because the thorax was well arched. Upon opening it, the lungs were found fully inflated almost covering the heart with its pericardium, the edges of all the lobes of the lungs well rounded. The lungs themselves with the heart attached may be said to have floated upon the water, more properly than in it. Blood flowed from the lungs when incised. The bladder was empty. There were spots of extravasation over the os frontis near the anterior fontanelle. I know that it is said that respiration is said to have taken place before the entire body is born. Such occurrences are however rare. It is doubtful whether the perfect inflation of the lungs, which existed in this case, could have taken place while the thorax was compressed by the outlet of the pelvis. The extravasations over the os frontis may, it is true, be supposed to have occurred after the death of the child, still they are more likely to have occurred before. But I do not know that the bladder has been found empty in any case where the child expired before delivery.

Did the child die from violence? The evidence upon this point is of two kinds, moral and circumstantial. Susan had constantly denied being pregnant—when seized with labor pains, she gave no notice; and after delivery, still denied the fact, and only admitted it after the placenta had been expelled.

It was in evidence that there was a great deal of blood upon the floor of the privy—that there was little or no blood in the vault of the privy. Here, a caution may be suggested, both as to the degree of blood upon the floor and as to the cause. Some persons use such terms as 'a great deal,' 'very little,' &c., quite loosely. But in this case, being used by the same person, they may be presumed to be used in ordinary relation to each other. This blood must have been discharged from the mother; and may have followed immediately upon the birth of the child; or if the child was indeed born whilst the mother was sitting on the seat of the privy, it may have escaped after she arose and was standing, considering what was to be done. It is to be regretted that the blood was hastily washed off without any observations having been made which would throw light upon the subject. Again, the vault of the privy though shallow was dark, and a candle was used in the search. Still the amount of blood about the child may not have been very correctly noted. It is to be remarked however, that the body of the child was not bloody. No record was made of the fact, but my recollection is, that there was no blood on the surface except on the scalp.

What caused the ecchymosis over the frontal bone? It could not have been occasioned, I should think, by falling on the feces and fluids of the vault. It might and probably was caused by falling upon the floor of the privy, from a greater or less height. The situation of the injury, it will be remarked is different from that considered usual from such falls, which is on one or both parietal bones. Could the complete inflation of the lungs have taken place whilst passing from the woman sitting on the seat to the place of deposit? I think not. I must acknowledge that my opinion is, that the child was born on the floor of the privy, and was thrown alive into the sink, where it perished by strangulation.

I am by no means satisfied with what I consider the indifference for human life manifested by the officers of the law in this case. Neither am I satisfied with the exposition of the law as reported in some similar cases, in Europe and America. I doubt the correctness of that exposition, as regards both justice and humanity. In the cases of *Rex. vs. Poulton: Same vs. Simpson: Rex vs. Sillis*, it was decided that the child must be wholly born from the mother alive, when violence was offered to it to justify a conviction for child murder; and Mr. Baron Parke thus charged the Grand Jury, at the Herts Lent Assizes in 1841: 'with respect to all these cases (of infanticide) there is a degree of doubt whether the infant has been *born* alive. The law requires that this should be *clearly proved* and that the whole body of the child should have come from the body of the parent. If it should appear that death was caused *during delivery*, then you will find a true bill.'—*Taylor's Med. Jur.* p. 377. In *Am. Jur. Med. Sciences*

vol. 17, p. 327 is a case reported by Dr. C. A. Lee of N. Y., in which the decision was rendered in conformity with the principle above quoted, with the marked approbation of the author—certainly high authority. With all respect however, I must enter my protest against the correctness of the Doctor's conclusions.

Whilst I should be exceedingly sorry to see a woman condemned for infanticide upon uncertain or insufficient evidence, and whilst I should cordially admit that a woman accused of infanticide, should be judged by the same rules and principles which are applied to others accused of murder, yet I do not see that those rules and principles should be made more lax and indefinite. Neither do I see any propriety for continuing a law upon the statute book which can never be enforced. Let us look at the matter. In the first place, the medical presumption and the legal presumption in the case are diametrically opposite. The former presumes a child born after seven months to be alive (*Taylor's Med. Jur.* p. 353.) The latter presumes it to be dead: and that too, although it may be proved beyond controversy, that only a very few moments before it was *entirely delivered*, it was alive. Dr. Lee defends the decision in the case above reported, on three grounds: 1st, The probability that the child had both breathed and died before it was born. He adduces a number of instances to prove that children had breathed before delivery was complete; and one from Baudeloque, in which the mouth of the child presenting at the os uteri, it cried before the head left the uterus. Of this last, Beck (*Med. Jur.* v. 1, p. 381) gives two additional cases. Of the very few cases in which respiration took place before the body of the child was born, a very small proportion indeed died before birth. Whether Baudeloque's case was born alive or dead, I do not know, but both cases quoted by Beck were born alive; one of them 'a full hour after it had cried in the womb.' It is I believe a settled point, that crying before complete delivery is not a source of *any danger* to the child. When death takes place under such a state, it is from the delay which would have proved fatal without respiration, and at the same time have prevented the birth being secret. Again 'in vagitus uterinus or vaginalis, the lungs receive a very small portion of air; in respiration after protrusion of the head, the lungs may be sometimes moderately well filled; although never, perhaps, possessing all the characteristic properties of those which have fully respired,' (*Taylor*, p. 375.)

2. The insufficiency of the Hydrostatic test. Under this Dr. L. makes two objections: *a.* That children had breathed and yet the lungs, whole, or cut into pieces, sank. This proves only what every medical jurist will readily admit, viz., that a feeble existence may be maintained for a while by an exceeding small portion of the lungs.—That in fact when cut into 'thirty pieces' (*Taylor* p. 366); just as if a man should put a grain of salt into a hogshead of water, and ask a chemist to detect its presence. *b.* That the lungs have been found to float in water when the child had not breathed, and when the lungs were neither putrid, emphysematous nor artificially inflated. He quotes several cases; two from *Bernt*, one from *Osiadel*, and three from *Am. Jour. Med. Science*, v. 8, p. 248. I have not been able to find the cases referred to, I do not find those from Bernt or Osiadel referred to by either Beck or Taylor, though Taylor does not quote Bernt to show that a child may breathe and yet the lung sink. His quotation from American Journal of Medical Sciences, vol. viii, p. 248 is certainly a wrong reference. I am not certain whether one of the cases from Bernt, and the one from Osiadel, are quoted from that work or not, but there is nothing of the kind there. Neither can I find them in that work. In vol. iv p. 248, is an extract from *Ouvrard* in which *Osiander* is quoted, but this only goes to show that the child may breathe and die before it is delivered. As I cannot find the cases so as to examine them in detail, and as Beck and Taylor do not refer to them, and as it is uncertain whether *Oscadel* is not a misprint for *Osiander*, who, as well as Bernt is quoted by them, I feel authorized in considering them as of no very great authority.

In opposition to this opinion of Dr. L., the elaborate examination of this subject by Beck and Taylor proves that the hydrostatic test is of very great importance in the investigation of infanticide; and also points out what it can, and what it cannot demonstrate. They likewise show that Dr. L's judgment that "In England and Germany the hydrostatic test is no longer considered conclusive. It will not be admitted in British courts, and ought not to be in ours;" is too hasty.

3. That the head of the child was fractured by falling upon the frozen ground, the birth having taken place whilst the woman was standing. That a woman may be delivered standing is not controverted—that a child thus born may have the skull fractured by falling on the floor or frozen ground is also probable. Upon this subject we may quote two extracts from Becks *Med. Jur.* Fifteen infants who had died before birth, but in whom there was no alteration in the bones of the cranium, were raised by the feet so that the head was about 18 inches from the floor, and suffered to fall perpendicularly. In twelve of them there was a longitudinal or angular fracture of one of the parietal bones, and sometimes of both, v. i. 399. "Of 183 sudden births, 155 children were expelled whilst the mothers were in the upright posture, 22 when sitting, and 6 when on the knees. Of the whole number not one child died; no fracture of the bones took place, nor any severe injury. Two only suffered temporary insensibility, and one an external wound with ecchymosis over the right parietal bone. i. p. 406. Dr. L. explains this exemption from injury thus: "It is highly probable that a large proportion of these cases occurred under the observation of the physicians who made the reports, and serious injury to the child was prevented by their interference. I look upon this supposition as not at all probable; for if the physician was present, he most likely would not have time to interfere. In Am. Jour Med. Sci. new series p. 270 is a case in which the child was suddenly expelled while the mother was standing on the floor. The funis was upturned about four inches from the body—there was no hemorrhage nor was the child at all injured by the fall.

Indeed I look upon the probability of injury to a child born whilst the mother is standing as much less than by falling eighten inches upon a floor or table. In the first place the mother will almost necessarily stoop so as to lessen the distance. Secondly the child is about twenty inches long and until entirely expelled it still affords some considerable resistence to the falling. Thirdly, even when the child is entirely expelled the umblical cord still affords some resistence—such indeed as sometimes to break the cord. These, I apprehend, are the true reasons of exemption from injury in such cases.

It is alleged, and alleged with propriety, that one accused of infanticide should be placed upon the same footing as one accused of homicide. That the same evidence should be required to convict of infanticide as is necessary to convict of homicide. In endeavoring to place the woman accused of infanticide, upon the same footing as for homicide, have we not put her upon other and materially different issues? Let us see. *The Queen v. Hagg.* Child found with a tape tied tightly round the neck. Full grown and healthy, had been alive as respiration had been fully established. The lungs filled the chest—floated on water and crepitated when pressed. From livid appearance of face and neck—congested state of the brain and extravasated blood on the surface, combined with the ligature round the neck, the witnesses were of opinion the child died of strangulation, * * one witness said the ligature had produced no mark on the neck, whilst others said it was perceptible. The counsel argued that a tape tied so tightly round the neck as to produce death, must have left a mark of which no person could have any doubt. The prisoner was convicted, Taylor 396. But she was convicted because her counsel mistook the ground upon which he ought to have defended her; as the next case proves. *Queen vs. Webster.* The child was full grown and was born alive:—this was inferred from the lungs being completely inflated. A ligature was found round the neck—it had been passed twice round—was very tight and fastened with a knot:—it had caused two deep indentations. The vessels of the brain and scalp were turgid with blood, but there were marks of external violence. Death was caused by strangulation. The judge left it to the jury to say whether they were satisfied that the child was wholly born into the world alive; and if so, whether the prisoner had not knowingly and wilfully destroyed it after it was born. The prisoner was acquitted. *Loco. cit.*

Just here, it may be well to mention decisions upon two points, somewhat different, but nearly allied to the above. In a case, in which the practitioner somehow fractured and otherwise injured the cranium, of which the child died soon after delivery, he was convicted of manslaughter *Id.* 398. In case of *Regina v. Reeves* it was decided that if a child be killed after it is completely delivered, but is still connected with the mother by means of the umbilcal cord, such killing is murder. *Am. Jour. Med. Sciences N. S.* v. 3 p. 246.

In the case reported by Dr. L., above referred to, the accused had always denied being pregnant—after delivery, denied the fact—afterwards acknowledged the birth—stated the child was not bigger than her fist—that she had buried it with a stick in the barn yard. The child was found wrapped in an old bag, behind and partly under the barn. It was a full grown male child—no external signs of violence except effused blood under the scalp covering the frontal bones—pericraneum separated from the bone—both parietal bones fractured, left in three places, right in one—chest externally arched—lungs described by one physician as collapsed and somewhat spungy—by another as filling the chest—about an inch square was cut from each lung, the pieces floated readily in both warm and cold water—that the injury to head was not inflicted after death—that it must have been done after birth with a stick or hard substance *On cross examination:* The injury of head could not have been produced by mother in attempts at self delivery. Child may be said to be born alive when it is furnished with an organization capable of supporting independent life—could not say that this child was born alive in that sense—the effusion of blood might have been caused by a blow shortly after death. Difficult, but not impossible for a woman to be delivered standing—it was possible the death of the child was occasioned by falling on the frozen ground. *For the prisoner.* There is no difficulty in delivery in the erect posture in quick labor, the woman being in street, this would be most probable—was probably the fact in the present case, as the fractures were in the very place we should expect to find them had they resulted from such an accident. Upon similar cases it may be well to remark the law, as interpreted, makes it utterly impossible to convict any person of infanticide, if that person is defended by counsel of tolerable ingenuity. It takes for granted a combination of facts which though not utterly impossible, it would defy the powers of computation to say how often it may happen. 1. That the woman was delivered suddenly; a thing of very rare occurrence. 2. That she was delivered standing; a thing, if any difference, still more rare. 3. That the child had breathed before it was completley born; another great rarity—in a practice of thirty-three years, I remember but one instance of it. 4. That after breathing and before birth is completed, or at any rate before the child reaches the ground, it has breathed sufficiently to inflate the lungs completely. 5. That the fall causes extensive fractures of the parietal bones. The almost impossible union of these 4 or 5 possibilities, some of which are incompatible with others, speedy birth, with death from imperfect respiration before birth, as also the full inflation of the lungs and death from same case, we are gravely asked to convert into a reasonable doubt of the prisoner's guilt!

But how stands the case of suspected homicide? Two men are known to have been near to each other upon an unfrequented spot; after which one disappears; the other is known to have reason, real or imaginary, to wish him out of the way. Upon being questioned, denies having seen him. Presently the missing man is found near the spot where they are known to have met, with marks of violence upon him, which could readily have been made by an instrument known to belong to the survivor. Will it avail the accused to say the commonwealth must prove that the deceased was actually alive when I got to him. I admit now that I did see him, but he was dead—my hostility to him was such that I mutilated his body after death? Yet this would be fully as reasonable as the decision in cases of infanticide. But take a real case. Videto, under the plea of having seen Indians about the house, borrowed a pistol and two guns. On the 1st of February, Mrs. Mosely lying in bed in the same room, about a yard from that occupied by Videto, with a curtain between, was shot in her sleep; of which wound she died in two hours. Videto stated that whilst he was watching, somebody thrust a gun in at the window suddenly and discharged it at Mrs. Mosely—that immediately he fired out at the window. The window was at the head of the bed, the ball entered the back near the hip, and came out near the left breast. Videto was convicted and executed. *Beck.* v. 2, p. 71.

Was his story any more unlikely than that a woman should tie a tape twice round the neck of her child after it had breathed and before it was born?

It may be blindness in me, but I must acknowledge my inability to perceive the propriety or the humanity of that law which permits a half born child to be killed with impunity, which in fact, considers such killing no crime: or the consistency of that law with one, which declares that injuring a child thus situated, in such manner as to cause its death soon after delivery, as highly criminal; (here killing is no crime, but maiming is a grievous one, ) or indeed, that which considers killing, when the child is born, but still attached to the mother by the umbilical cord, as murder. It occasionally happens that the debility of the child requires the cord to be kept entire for a short time. In a case of this kind, the child not being able to support an independent existence, killing is no murder. We have to act only upon the recognized principle, and presume *every child* to be incapable of supporting an independent existence,* until the contrary is *clearly proved*, and the last remnant, (if indeed there is any remnant,) of a protection of the child's life is taken away.

In our solicitude to protect the rights and life of the mother, we have entirely lost sight of those of the child. It is to be hoped, that this whole matter will be speedily revised by our legislatures, and principles established, more consonant with justice, humanity and common sense.

In conclusion, I may make a remark, that as any physician may at any moment be required to attend a coroner's inquest and testify as to the causes of death, how many of us are prepared to determine at a moment's notice, the great variety of questions which may arise on the occa-

---

* [NOTE.] This is no more repugnant to reason than that a child of common size whose lungs have been perfectly inflated and having marks of violence upon it was *born* dead.

sion? How many of us are qualified when thus suddenly called on, so to discharge our duty as to screen the innocent from a cruel and unjust suspicion, and so to develop circumstances as to direct attention to the guilty, though in fancied security? We should be on our guard too, that whilst we admit the possibility of a fact, it be so done that an expert counsel shall not make the impression that the fact is of every day's occurence. My own opinion is, that some one physician should be designated in each county, whose duty it should be to qualify himself in an especial manner for this duty, and who should in all cases be called on.

I sat down to give the facts of the case of the negro woman Susan; and find I have written almost an essay on infanticide, law, and divers other things. If, however, I shall excite a few to look more carefully into the subject of Medical Jurisprudence I shall not have lost my labor.

GEORGETOWN, NOV. 15, 1852.

